# A long non-coding RNA signature to improve prognosis prediction of gastric cancer

**DOI:** 10.1186/s12943-016-0544-0

**Published:** 2016-09-20

**Authors:** Xiaoqiang Zhu, Xianglong Tian, Chenyang Yu, Chaoqin Shen, Tingting Yan, Jie Hong, Zheng Wang, Jing-Yuan Fang, Haoyan Chen

**Affiliations:** 1 Division of Gastroenterology and Hepatology, Key Laboratory of Gastroenterology and Hepatology, Ministry of Health, State Key Laboratory for Oncogenes and Related Genes, Renji Hospital, School of Medicine, Shanghai JiaoTong University, Shanghai Institute of Digestive Disease, 145 Middle Shandong Road, Shanghai, 200001 China; 2 Department of gastrointestinal surgery, Renji Hospital, School of Medicine, Shanghai Jiao Tong University, Shanghai, 200127 China

**Keywords:** Gastric cancer, LncRNAs, LNR, GSEA, Survival

## Abstract

**Background:**

Increasing evidence suggests long non-coding RNAs (lncRNAs) are frequently aberrantly expressed in cancers, however, few related lncRNA signatures have been established for prediction of cancer prognosis. We aimed at developing alncRNA signature to improve prognosis prediction of gastric cancer (GC).

**Methods:**

Using a lncRNA-mining approach, we performed lncRNA expression profiling in large GC cohorts from Gene Expression Ominus (GEO), including GSE62254 data set (*N =* 300) and GSE15459 data set (*N =* 192). We established a set of 24-lncRNAs that were significantly associated with the disease free survival (DFS) in the test series.

**Results:**

Based on this 24-lncRNA signature, the test series patients could be classified into high-risk or low-risk subgroup with significantly different DFS (HR = 1.19, 95 % CI = 1.13–1.25, *P* < 0.0001). The prognostic value of this 24-lncRNA signature was confirmed in the internal validation series and another external validation series, respectively. Further analysis revealed that the prognostic value of this signature was independent of lymph node ratio (LNR) and postoperative chemotherapy. Gene set enrichment analysis (GSEA) indicated that high risk score group was associated with several cancer recurrence and metastasis associated pathways.

**Conclusions:**

The identification of the prognostic lncRNAs indicates the potential roles of lncRNAs in GC biogenesis. Our results may provide an efficient classification tool for clinical prognosis evaluation of GC.

**Electronic supplementary material:**

The online version of this article (doi:10.1186/s12943-016-0544-0) contains supplementary material, which is available to authorized users.

## Background

Being the fourth most common malignancy, GC has been the second leading cause of cancer deaths worldwide [1]. An estimated 951,600 GC cases occurred and 723,100 patients died from GC in 2012 [1, 2]. Adequate surgical resection is the only curative therapeutic option for GC [3, 4]. The current strategy to GC management, which has significantly improved overall survival (OS) [4], includes endoscopic detection followed by gastrectomy and chemotherapy or chemo-radiotherapy in neoadjuvant or adjuvant regiments [5]. However, treatment outcome still remains undesirable. The current Union International Committee on Cancer (UICC) or the American Joint Committee on Cancer (AJCC) TNM stage system has shown valuable but insufficient prediction for prognosis and estimation for subsets of GC patients [6–8]. An increasing amount of evidence demonstrates that the discovery and application of molecular biomarkers will promote the prognostic evaluation and identification of potential high-risky GC patients [5, 9, 10].

Currently, with the advancements in transcriptome profiling, the roles of dysregulated functional long non-coding RNAs (lncRNAs) in human cancers have received considerable attention [11–13]. LncRNAs are mRNA-like transcripts ranging in length from 200 nucleotides (nt) to ~ 100 kilobases (kb) that lack significant protein-coding abilities [14, 15]. Increasing evidence suggests that the aberrant expressions of lncRNAs have been associated with human cancers [16–18], and some of them have been implicated in diagnosis and prognostication [19, 20]. Although several prognostic biomarkers for GC have been undergoing or tested in clinical trials such as Fibroblast Growth Factor Receptor (FGFR) [21], Human Epidermal Growth Factor Receptor 2 (HER2) [22], Epidermal Growth Factor Receptor (EGFR) [23], Hepatocyte Growth Factor Receptor (HGFR) [24], etc, many more potential and valuable molecular biomarkers are urgent to be discovered and identified to improve the clinical outcome of patients with GC. Increasing studies have shown that lncRNAs could be one of the best candidates as potential prognostic biomarkers in GC [25–27]. Therefore, searching a lncRNA signature might be concrete predictive and prognostic value in the management of GC.

However, lncRNA profiles in most human cancers remain largely unknown, mainly due to the lack of such arrays. Previous studies have demonstrated that lncRNA profiling could be achieved by mining previously published gene expression microarray data because a large amount of lncRNA-specific probes were fortuitously represented on the commonly used microarray platforms [28, 29]. In the present study, we applied this method to conduct gene expressions of lncRNAs profiling on a cohort of 300 patients from GSE62254 as well as another independent data set from GEO database. By using the sample-splitting method, random survival forests-variable hunting (RSF-VH) algorithm and Cox regression analysis, we identified a prognostic, 24-lncRNA signature from the GSE62254 test series patients, and validated it in the GSE62254 validation series and another independent GEO cohort (GSE15459).

## Methods

### GC datasets preparation

Microarray data from GSE62254 and GSE15459 data sets were directly downloaded from Gene Expression Omnibus (http://www.ncbi.nlm.nih.gov/geo/). These datasets corresponded to all available public datasets fulfilling the following criteria: available gene expression data obtained using the same chip platform (Affymetrix Human Genome U133 Plus 2.0 chips) with raw data CEL files, and patient outcome data available. After initial quality check, two panels of GC gene expression data sets were included in our study: GSE62254 and GSE15459. The GC samples in GSE62254 were randomly split into a test series (*n =* 180) and an internal validation series (*n =* 120). Additionally, the GC samples in GSE15459 were analyzed as an external validation series.

### Microarray data processing and lncRNA profile mining

The raw CEL files were downloaded from GEO database and background adjusted using Robust Multichip Average (RMA) [30] which has been shown to be a solid measure tool for lncRNA profiling data [31]. The approach of lncRNA profile mining mainly referred to Xiaoqin Zhang et al [32]. Briefly, we mapped the Affymetrix HG-U133 Plus 2.0 probe set IDs to the NetAffx Annotation Files. Based on the Refseq transcript ID and/or Ensembl gene ID in NetAffx annotations, we only retained non-coding protein genes and further filtered them by eliminating pseudogenes including microRNAs, rRNAs and other short RNAs such as snoRNAs, snRNAs and tRNAs. Finally, 2448 annotated lncRNA transcripts with corresponding Affymetrix probe IDs were generated.

### GSEA

GSEA was performed by the JAVA program (http://software.broadinstitute.org/gsea/index.jsp) using MSigDB C2 CP: Canonical pathways gene set collection. The GSEA, visualized in Cytoscape (version 2.8.0) was used to determine whether the members of a given gene set were generally associated with risk score, and was therefore conducted on all mRNA genes on the HG-U133 Plus 2.0 ranked by enrichment score from most positive and most negative. 1000 random sample permutations were carried out, and the significance threshold set at FDR < 0.01. If a gene set had a positive enrichment score, the majority of its members had higher expression accompanied with higher risk score, and the set was termed “enriched”.

### Bioinformatics analysis

All statistical analyses were conducted using R software [33] and Bioconductor [34]. The association between the lncRNA expression and patient’s DFS or OS was assessed by univariable Cox regression analysis along with a permutation test using BRB-Array Tools [35]. The permutation *p*-values for significant genes were computed based on 10,000 random permutations and genes were considered statistically significant if their permutation *p* values were less than 0.01. And genes that passed the filter criteria were considered for further analysis by applying the random survival forest-variable hunting (RSF-VH) algorithm [36]. Among the parameters involving in this algorithm, the number of nsplit was set as nsplit = 10 following Ishwaran and colleagues [37] in the variable selection function within the Random Survival Forest package during the selection. To construct a predictive model, the candidate genes were fitted in a univariable Cox regression model in the test series as previously applied [38]. A risk score formula was then established by including each of these selected genes, weighted by their estimated regression coefficients in the univariable Cox regression analysis [38]. With this risk score formula, patients in each set were classified into high-risk or low-risk group by using the corresponding median risk score as the cutoff point. Survival differences between the high-risk and low-risk groups in each set were assessed by the Kaplan-Meier estimate, and compared using the log-rank test. To test whether the risk score was independent of LNR and postoperative chemotherapy, multivariable Cox regression and data stratification analysis were performed. We performed ROC analysis to compare the sensitivity and specificity of the survival prediction based on the lncRNA risk score, AJCC stage, LNR and postoperative chemotherapy. To generate ROC curves, patients were classified as surviving either longer or shorter than the median DFS, excluding patients who were alive for durations less than the median DFS at last follow-up [39]. In the log-rank test, Cox regression analysis and ROC analysis, the significance was defined as *P* values being less than 0.05.

## Results

### GC data sets preparation

GC data sets and corresponding clinical data were downloaded from the publicly available GEO database. The following two cohorts of GC gene expression data were included in this study: GSE62254 [5] and GSE15459 [40]. After removal of the samples without survival status, a total of 492 GC patients analyzed in the present study (see Additional file 1). These included 300 GC patients from GSE62254 (180 patients from the test series and 120 patients from the validation series). And 192 GC patients from GSE15459 were included after 8 patients were removed due to absence of clinical outcome information.

### Identification of prognostic lncRNA genes from the test series

The 300 GC samples were randomly assigned to a test series (*n =* 180) or a validation series (120). The test series was used for the detection of prognostic lncRNA genes. By subjecting the lncRNA expression data of the test series to univariable Cox regression proportional hazards regression analysis using Biometric Research Branch-Array (BRB-Array) Tools, we identified a set of 63 genes whose parameter *P*-value were less than 0.01. Those 63 genes were further analyzed by random survival forest-variable hunting (RSF-VH) algorithm [36]. This algorithm is a high-dimensional order statistic measuring the predictiveness of a variable in a survival tree that exploits maximal subtrees for effective variable selection under such scenarios [36]. With this method, 24 genes were screened out as the predictors (genes). Table 1 showed a list of genes with their obtained specific values including permutation *P* values, hazard ratios and coefficients which of these were derived from the univariable Cox proportional hazards regression analysis. Moreover, the variable importance values were also figured out following the variable selection function within the Random Survival Forest package. Variable importance measures the increase (or decrease) in the prediction error for the random forests model when a variable is randomly “noise up”. That is if the prediction error of the model became worse when the effect of a variable in the model on the prediction was intentionally destroyed, this means that the variable is important in the model [41, 42]. Among these genes, positive coefficients indicated that the higher expression levels of 14 genes (AF035291, AI028608, AK026189, H04858, BC037827, BC038210, AI916498, AA463827, AA041523, BE621082, AK056852, AW206234, AL703532, AI095542) were associated with shorter survival. The negative coefficients for the remaining ten genes (AI080288, BC021187, BF238392, BC005107, BC039674, AI056187, T79746, H11436, BF511694, BC035722) indicated that their higher levels of expression were associated with longer survival.

**Table 1:** LncRNAs significantly associated with the disease free survival in the test series patients (*N =* 180)

Probe	Gene symbol	Permutation *P* value^a,b^	Hazard ratio^a^	Coefficient^a^	VI	Associated diseases	Description
233512_at	AF035291	0.0017	8.325	2.11846	0.012	NR	hypothetical LOC100287216
1568854_at	AI028608	0.0084	6.839	1.92247	−0.002	NR	non-protein coding RNA 240
229280_s_at	AK026189	6.00E-04	4.633	1.53266	0.006	Melanoma and basal cell carcinoma	promotes metastasis and recurrence of melanoma
1559412_at	H04858	8.00E-04	4.030	1.3926	0.002	Acute megakaryoblastic leukemia	Maintenance of leukemic growth
1559965_at	BC037827	0.0018	3.587	1.27718	0.053	NR	hypothetical LOC100192378
1557338_x_at	BC038210	0.0108	3.326	1.20171	0.017	NR	NR
230589_at	AI916498	0.0034	2.844	1.04591	−0.006	Gastric cancer	Sensitivity of iodine-125 particle irradiation and regulation of NF-KB signaling pathway
239466_at	AA463827	0.0013	2.799	1.0294	0.026	NR	hypothetical LOC344595
230251_at	AA041523	0.0014	2.521	0.92436	0.038	Non-small cell lung cancer	Associated with loss function of LKB1 gene
225029_at	BE621082	0.0016	2.249	0.81047	0.007	Systemic lupus erythematosus	A novel susceptibility locus on Xp11.21
1564139_at	AK056852	0.0016	1.923	0.65369	0.004	NR	hypothetical LOC144571
229014_at	AW206234	0.0021	1.718	0.54056	0.002	NR	hypothetical LOC441094
1558828_s_at	AL703532	0.0032	1.325	0.2811	0.005	Heart failure associated diseases	Regulation of cardiac cell differentiation and homeostasis
235759_at	AI095542	0.0034	1.282	0.24825	−0.004	NR	NR
213972_at	AI080288	0.0036	0.155	−1.86125	−0.007	NR	NR
1554880_at	BC021187	0.0024	0.106	−2.24862	−0.002	Gastric cancer	Protective roles for gastric cancer
235824_at	BF238392	0.0019	0.073	−2.61423	0.012	NR	NR
232191_at	BC005107	0.0076	0.070	−2.65478	0.002	NR	chromosome 21 open reading frame 105
1563296_at	BC039674	0.0022	0.068	−2.69258	0.000	NR	NR
239617_at	AI056187	9.00E-04	0.061	−2.79863	−0.001	Gastric cancer	spans the promoter and untranslated regions\of the ghrelin gene
243975_at	T79746	0.0031	0.058	−2.85076	0.003	NR	NR
1558666_at	H11436	3.00E-04	0.055	−2.89127	−0.007	NR	NR
237471_at	BF511694	0.0034	0.041	−3.19733	0.003	NR	hypothetical LOC154822
1562683_a_at	BC035722	0.0023	0.033	−3.40924	0.001	NR	hypothetical LOC285547

*Abbreviations*: *VI* Variable Importance, *NR* Not Reported

^a^Derived from the univariable Cox proportional hazards regression analysis in the 180 test series patients

^b^Obtained from permutation test repeated 10,000 times

### The 24-lncRNA signature and the patients’ survival in the test series

**Fig. 1:**
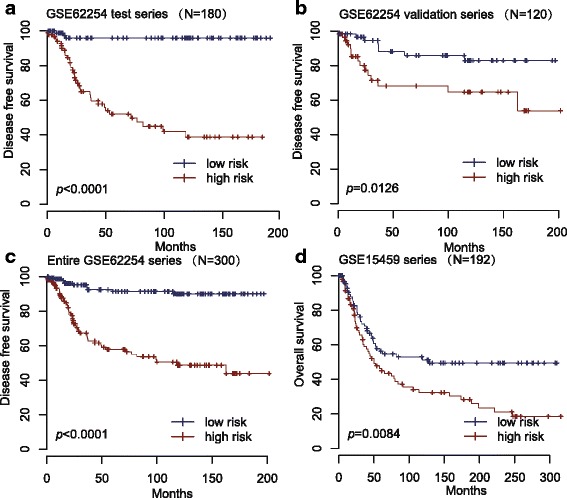
Kaplan-Meier estimates of the disease free survival (DFS) or overall survival (OS) of GEO patients using the 24-lncRNA signature. The Kaplan-Meier plots were used to visualize the DFS probabilities for the low-risk versus high-risk group of patients based on the median risk score from corresponding GEO datasets patents. **a** Kaplan-Meier curves for GSE62254 test series patients (*N =* 180); (**b**) Kaplan-Meier curves for GSE62254 validation series patients (*N =* 120); (**c**) Kaplan-Meier curves for the entire GSE62254 series patients (combined test and validation series patients, *N =* 300). **d** Kaplan-Meier curves for GSE15459 patients (*N =* 192). The tick marks on the Kaplan-Meier curves represent the censored subjects. The differences between the two curves were determined by the two-side log-rank test

**Fig. 2:**
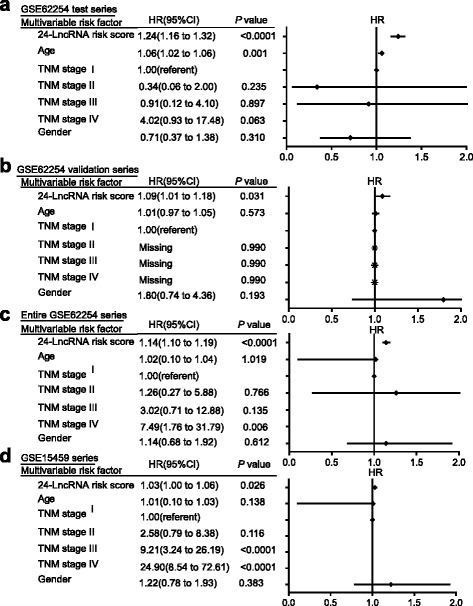
Comparison of the score with prognostic clinical covariates. Multivariable Cox regression proportional hazards regression analyses incorporating the risk score and known prognostic clinical factors, including age at diagnosis, TNM stage (I, II, III, IV) and gender; risk score and age as continuous variables, TNM stage and gender as categorical variables. Solid tetragonums represent the HR of death and open-ended horizontal lines represent the 95 % confidence intervals (CIs). All *P* values were calculated using Cox proportional hazards analysis. **a** Multivariable analysis was performed using Cox proportional hazards regression analysis in patients of GSE62254 test series. **b** Multivariable analysis was performed using Cox proportional hazards regression analysis in patients of GSE62254 validation series. **c** Multivariable analysis was performed using Cox proportional hazards regression analysis in patients of entireGSE62254 series. **d** Multivariable analysis was performed using Cox proportional hazards regression analysis in patients of GSE15459 series. All of these were adjusted for the same categorical or continuous variables. Missing: HR (95 % CI) could not be calculated out

A risk-score formula was created based on the expression of these 24 lncRNAs for DFS prediction, as follows: Risk score = (2.11846*expression level of AF035291) + (1.92247*expression level of AI028608) + (1.53266*expression level of AK026189) + (1.3926* expression level of H04858) + (1.27718* expression level of BC037827) + (1.20171* expression level of BC038210) + (1.04591* expression level of AI916498) + (1.0294* expression level of AA463827) + (0.92436* expression level of AA041523) + (0.81047* expression level of BE621082) + (0.65369* expression level of AK056852) + (0.54056* expression level of AW206234) + (0.2811* expression level of AL703532) + (0.24825* expression level of AI095542) + (-1.86125* expression level of AI080288) + (-2.24862* expression level of BC021187) + (-2.61423* expression level of BF238392) + (-2.65478* expression level of BC005107) + (-2.69258* expression level of BC039674) + (-2.79863* expression level of AI056187) + (-2.85076* expression level of T79746) + (-2.89127* expression level of H11436) + (-3.19733* expression level of BF511694) + (-3.40924* expression level of BC035722). We then calculated the 24-lncRNA signature risk score for each patient in the test series, and ranked them according to their risk scores. As such, patients were divided into a high-risk group (*n =* 90) or a low-risk group (*n =* 90) using the median risk score of the test series as the cutoff point. Patients in the high-risk group had significantly shorter median DFS than those in the low-risk group (log-rank test *P* < 0.0001) (Fig. 1a). The association of the 24-lncRNA risk score with DFS was also significant when it was evaluated as a continuous variable in the multivariable Cox regression model (Fig. 2a).

### Validation of the 24-lncRNA signature for survival prediction in the validation series and the entire GSE62254 data set

To confirm our findings, we validated our 24-lncRNA signature in the internal validation series. By using the same risk formula, we classified patients into high-risk (*n =* 60) or low-risk group (*n =* 60) using the median score of the internal validation series as the cutoff point. In the consistence with the findings described above, patients in the high-risk group had significantly shorter median DFS than those in the low-risk group (log-rank test *P* = 0.0126) (Fig. 1b). Risk score-based classification of the entire GSE62254 cohort (i.e. combined test and validation series) also yielded similar results (log-rank test *P* < 0.0001) (Fig. 1c). In the multivariable Cox regression model that the 24-lncRNA risk score was evaluated as a continuous variable, similar correlation could be observed (Fig. 2b-c).

**Fig. 3:**
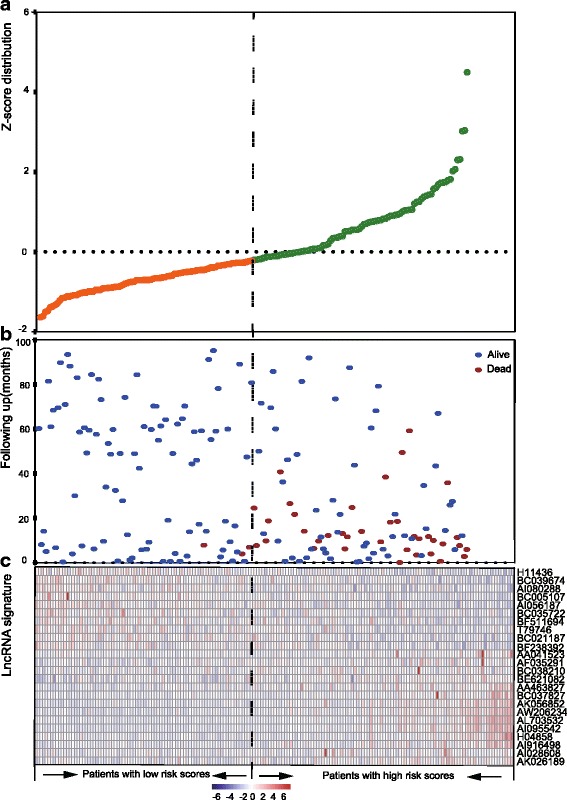
LncRNA risk score analysis of GSE62254 test series. The distribution of 24-lncRNA risk score, patients’ survival status and lncRNA expression signature were analyzed in the GSE62254 test series patients (*N =* 180). **a** lncRNA signature risk score distribution; (**b**) patients’ survival status and time; (**c**) heatmap of the lncRNA expression profiles. Rows represent lncRNAs, and columns represent patients. The black dotted line represents the median lncRNA risk score cutoff dividing patients into low-risk and high-risk groups

The distribution of the lncRNA risk score, the survival status of the GC patients and the lncRNA expression signature were also obtained. As shown in the Fig. 3, in the GSE62254 test series patients, we found that patients with high-risk scores tended to express high level of risky lncRNAs (AF035291, AI028608, AK026189,H04858,BC037827, BC038210, AI916498, AA463827, AA041523, BE621082, AK056852, AW206234, AL703532, AI095542) in their tumors, whereas patients with low-risk scores tended to express high level of protective lncRNAs (AI080288, BC021187, BF238392, BC005107, BC039674, AI056187, T79746, H11436, BF511694, BC035722).

### Further validation of the 24-lncRNA signature in another independent data set

We further validated our 24-lncRNA signature in another independent GC data set obtained from GEO, GSE15459. The clinical characteristics of this cohort were also listed (see Additional file 1). Although the patient outcome was represented with OS, this data set confirmed the ability of our model in predicting survival. As shown in Fig. 1d, the 24-lncRNA model could effectively predict the OS in patients from GSE15459 (log-rank test *P* = 0.0084). In the multivariable Cox regression model, the lncRNA risk score was significantly associated with OS as a continuous variable in the GSE15459 cohort (Fig. 2d).

### Prognostic value of the 24-lncRNA signature is independent of LNR

**Fig. 4:**
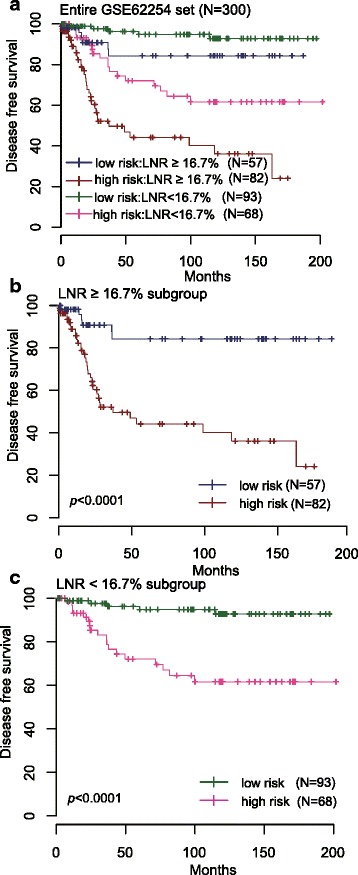
Kaplan-Meier estimates of the disease free survival (DFS) of GEO patients using the 24-lncRNA signature, stratified by lymph node ratio (LNR). Entire GSE62254 set (*N =* 300) were first stratified by LNR (LNR ≥ 16.7 % or LNR < 16.7 %). Kaplan-Meier plots were then used to visualize the survival probabilities for the high-risk versus low-risk group of patients determined on the basis of the median risk score from the entire GSE62254 set patients within each LNR stratum. **a** Kaplan-Meier curves for the entire GSE62254 set patients (*N =* 300); (**b**) Kaplan-Meier curves for patients LNR ≥16.7 % (*N =* 139); (**c**) Kaplan-Meier curves for patients LNR < 16.7 % (*N =* 161). The tick marks on the Kaplan-Meier curves represent the censored subjects. The differences between the two curves were determined by the two-sided log-rank test

LNR is the ratio of the numbers of metastatic lymph modes to those of the dissected lymph nodes. Increasing evidence indicated that LNR is a novel and simple marker which can easily stratify the prognoses of advanced GC [43–45]. And several studies have demonstrated that LNR = 16.7 % was the optimal cutoff level as an effective prognostic indictor in advanced GC [46, 47]. Fortunately, LNR could be calculated out in GSE62254 data set for 300 patients. Thus, we tested whether the prognostic value of the 24-lncRNA signature was independent of LNR. For this, we first conducted multivariable Cox regression analysis and stratification analysis. In the multivariable Cox regression analysis on these 300 patients that contained 24-lncRNA risk score, LNR, age and gender as covariates, we found that the 24-lncRNA risk score (HR = 1.17, 95 % CI = 1.12–1.23, *P* <0.0001) and LNR (HR = 12.63, 95 % CI = 4.90–32.60, *P* < 0.0001) were both independent prognostic factors (Table 2). Data stratification analysis was then performed which stratified these patients into LNR ≥ 16.7 % subgroup and LNR < 16.7 % subgroup. The stratification analysis showed that the 24-lnsRNA signature could identify patients with different prognoses despite of the same LNR stratum (Fig. 4a). For instance, among the patients LNR ≥ 16.7 % (*n =* 139), the 24-lncRNA risk score could further subdivide them into those likely to have longer versus shorter survival (log-rank test *P* <0.0001) (Fig. 4b). Similarly, among those LNR < 16.7 % (*n =* 161), the 24-lncRNA risk score could also subdivide patients into two subgroups with significantly disparate survival (log-rank test *P* < 0.0001) (Fig. 4c).

**Table 2:** Multivariable Cox regression analysis of the 24-lncRNA risk score, LNR and postoperative chemotherapy in GSE62254 series

Variables	HR	95 % CI of HR	*P* value
24-lncRNA risk score (*N =* 300)	1.16	1.10 –1.21	<0.0001
LNR	4.33	1.34 –13.93	0.014
Age	1.03	1.00 –1.05	0.032
Gender	1.10	0.65 –1.84	0.732
TNM stage I	1.00(referent)		
TNM stage II	1.08	0.23 –5.08	0.919
TNM stage III	2.09	0.47 –9.24	0.330
TNM stage IV	3.89	0.82 –18.44	0.087
24-lncRNA risk score (*N =* 299)	1.14	1.09 –1.19	< 0.0001
Postoperative chemotherapy	0.36	0.18 –0.73	0.004
Age	1.01	0.99 –1.03	0.324
Gender	1.04	0.62 –1.75	0.897
TNM stage I	1.00(referent)		
TNM stage II	1.47	0.31 –6.85	0.626
TNM stage III	4.00	0.93 –17.15	0.062
TNM stage IV	8.50	2.00 –36.20	0.004

In Cox regression analysis, risk score, LNR and age were evaluated as continuous variables, and postoperative chemotherapy and gender were evaluated as category variables

*Abbreviations*: *LNR* lymph node ratio, *HR* hazard ratio

### Prognostic value of the 24-lncRNA signature is independent of postoperative chemotherapy

**Fig. 5:**
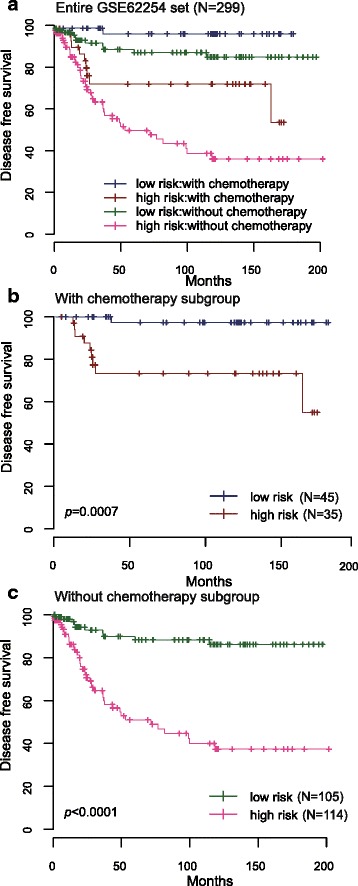
Kaplan-Meier estimates of the disease free survival (DFS) of GEO patients using the 24-lncRNA signature, stratified by postoperative chemotherapy. Entire GSE62254 set (*N =* 299) were first stratified by postoperative chemotherapy (with or without postoperative chemotherapy). Kaplan-Meier plots were then used to visualize the survival probabilities for the high-risk versus low-risk group of patients determined on the basis of the median risk score from the GSE62254 set patients within each postoperative chemotherapy stratum. **a** Kaplan-Meier curves for the entire GSE62254 set patients (*N =* 299); (**b**) Kaplan-Meier curves for patients with postoperative chemotherapy (*N =* 80); (**c**) Kaplan-Meier curves for patients without postoperative chemotherapy (*N =* 219). The tick marks on the Kaplan-Meier curves represent the censored subjects. The differences between the two curves were determined by the two-sided log-rank test

We also tested whether the prognostic value of the 24-lncRNA signature was independent of postoperative chemotherapy. To achieve this, we first conducted multivariable Cox regression analysis and stratification analysis. By inspection, of the 300 GSE62254 samples analyzed, 299 patients had available data on their postoperative chemotherapy information. Unfortunately, of the 192 patients from GSE15459, no patients had available information on their postoperative chemotherapy. In the multivariable Cox regression analysis on these 299 patients that contained 24-lncRNA risk score, postoperative chemotherapy, age and gender as covariates, we found that the 24-lncRNA risk score (HR = 1.17, 95 % CI = 1.13–1.22, *P* < 0.0001) and postoperative chemotherapy (HR = 0.38, 95 % CI = 0.19–0.76, *P* = 0.0060) were both independent prognostic factors (Table 2). Data stratification analysis was then performed which stratified these patients into with postoperative chemotherapy subgroup or without postoperative chemotherapy subgroup. The stratification analysis showed that the 24-lncRNA signature could identify patients with different prognoses despite of the same postoperative chemotherapy stratum (Fig. 5a). For instance, among the patients with postoperative chemotherapy (*n =* 80), the 24-lncRNA risk score could further subdivide them into those likely to have longer versus shorter survival (log-rank test *P* = 0.0007) (Fig. 5b). Similarly, among those without postoperative chemotherapy (*n =* 219), the 24-lncRNA risk score could still subdivide patients into two subgroups with significantly disparate survival (log-rank test *P* < 0.0001) (Fig. 5c).

### Prognostic value of the 24-lncRNA signature is independent of TNM stage

**Fig. 6:**
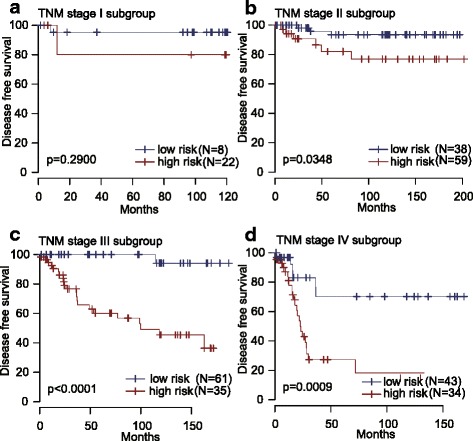
Kaplan-Meier estimates of the disease free survival (DFS) of GEO patients using the 24-lncRNA signature, stratified by TNM stage (I, II, III & IV). Kaplan-Meier plots were then used to visualize the survival probabilities for the high-risk versus low-risk group of patients determined on the basis of the median risk score from the entire GSE62254 set patients within each TNM stage. **a** Kaplan-Meier curves for patients with TNM stage I (*N =* 30); (**b**) Kaplan-Meier curves for patients with TNM stage II (*N =* 97); (**c**) Kaplan-Meier curves for patients with TNM stage III (*N =* 96); (**d**) Kaplan-Meier curves for patients with TNM stage IV (*N =* 77). The tick marks on the Kaplan-Meier curves represent the censored subjects. The differences between the two curves were determined by the two-sided log-rank test

According to TNM stage system for GC, patients in GSE62254 series were divided into four subgroups (I, II, III and IV). The stratification analysis suggested that the 24-lncRNA signature could identify patients with different prognoses in each TNM stage subgroup (Fig. 6a-d) despite that the *P* value was not significant in stage I (log-rank test *P* = 0.2900). This might be because the sample size was too small (only 30 patients, Fig. 6a) to draw any reliable conclusions. Interestingly, when low TNM stage (I & II) and high TNM stage (III & IV) were combined, respectively, the 24-lncRNA signature could also identify patients with different prognoses in each subgroup and the *P* value was significant (see Additional file 2).

**Fig. 7:**
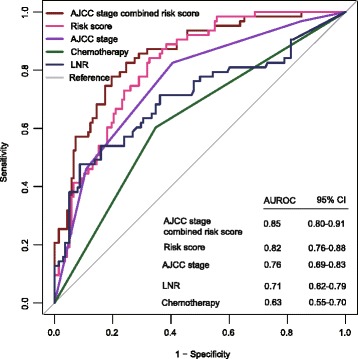
Receiver operating characteristic(ROC) analysis of the sensitivity and specificity of the disease free survival (DFS) prediction by the 24-lncRNA risk score, AJCC stage, lymph node ratio (LNR) and postoperative chemotherapy in GSE62254 set patients with known chemotherapy information (*N =* 202). *P* values were from the comparisons of the area under the ROC (AUROC) of 24-lncRNA risk score versus those of AJCC stage, 24-lncRNA risk score combined with AJCC stage, LNR and postoperative chemotherapy, respectively. As can be seen, the 24-lncRNA risk score combined with AJCC stage showed a better prediction of DFS than AJCC stage (*P* = 0.0002). The predictive ability of risk score was equivalent to AJCC stage alone (*P* = 0.1861), but better than both LNR (*P* = 0.0297) and postoperative chemotherapy (*P* < 0.0001)

Additionally, we performed ROC analysis to compare the sensitivity and specificity of survival prediction among the 24-lncRNA risk score model, AJCC stage, LNR and postoperative chemotherapy. The area under receiver operating characteristic (AUROC) was assessed and compared among the four prognostic factors. As shown in Fig. 7, there was no significant difference between the AUROC of 24-lncRNA risk score when compared with AJCC stage (0.82 versus 0.76, 95 % CI = 0.76–0.88, *P* = 0.1861). However, the AUCROC of the 24-lncRNA signature risk score combined with AJCC stage was significantly greater than AJCC stage alone (0.85 versus 0.76, 95 % CI = 0.69–0.83, *P* = 0.0002). Additionally, the 24-lncRNA signature risk score was significantly superior than that of LNR (0.82 versus 0.71, 95 % CI = 0.62–0.79, *P* = 0.0297) and postoperative chemotherapy (0.82 versus 0.63, 95 % CI = 0.55–0.70, *P* < 0.0001). Although the predictive ability of the 24-lncRNA signature was equivalent to AJCC stage, these results also indicated that the 24-lncRNA signature combined with AJCC stage may have a stronger power for DFS prediction in the ROC analysis. Also, the 24-lncRNA signature may have a better predictive ability than both LNR and postoperative chemotherapy alone.

### Identification of 24-lncRNA signature correlated biological pathways and processes

**Fig. 8:**
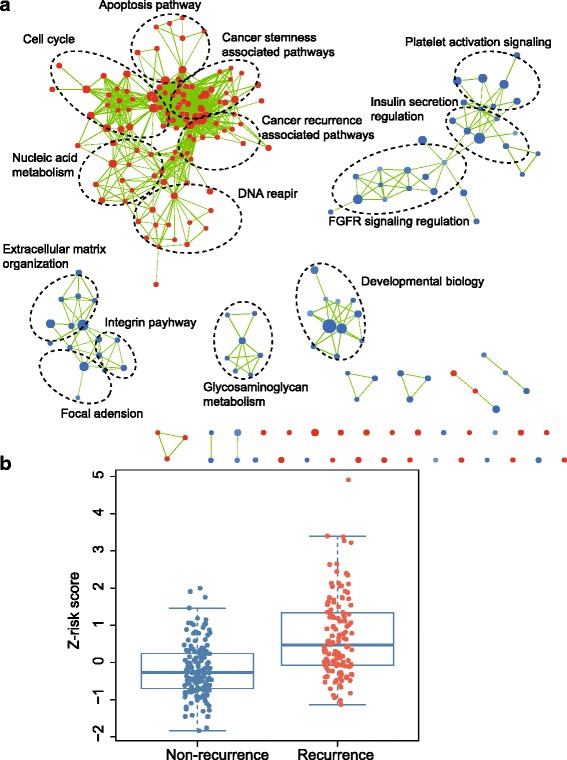
**a** Gene set enrichment analysis delineates biological pathways and processes correlated with risk score. Cytoscape was used for visualization of the GESA results. Nodes represent enriched gene sets that are grouped and annotated by their similarity according to related gene sets. Enrichment results were mapped as a network of gene sets (nodes). Node size is proportional to the total number of genes within each gene set. Proportion of shared genes between gene sets is represented as the thickness of the green line between nodes. **b** Box plot of risk score of patients with or without recurrence in entire GSE62254 series excluding patients without available information (*N =* 283, *P* < 0.0001).*T*-test was used to determine the significance of the comparisons

We performed GSEA to identify correlated biological process and signaling pathways using the 24-lncRNA signature on the basis of risk score for classification. Significant gene sets (FDR < 0.001, *P* < 0.05) were visualized as interaction networks with Cytoscape (Fig. 8a, see Additional file 3). The high risk score was accompanied with up-regulation of several cancer-related networks including recurrence, metastasis and cancer stemness associated pathways. For instance, Polo-like kinase 1 (PLK1) and E2F-mediated associated pathways were implicated in cancer recurrence and metastasis [48–50]. We proposed that the 24-lncRNA signature might be involved in these networks. Since cancer recurrence and metastasis could strongly affect patients’ DFS, we compared the risk score of patients with recurrence and without recurrence (non-recurrence) in GSE62254 series when this information was available. Patients with recurrence tended to have higher risk score than patients without recurrence (Fig. 8b, *P* < 0.0001).

## Discussion

Currently, the discovery of thousands of lncRNAs has broken the conventional thinking that the gene regulation in biology was mostly involved in protein-coding genes [51, 52]. Evidence from growing publications have demonstrated that functional lncRNAs expression patterns were associated with human cancers [11–14]. These lncRNAs were implicated in various tumorigenesis processes including proliferation [53], invasion [54] and apoptosis [55] by acting as tumor oncogenes or suppressors. The aberrant expressions of specific lncRNAs in cancer can mark the spectrum of disease progression and may serve as independent biomarkers for diagnosis and prognosis [29, 32]. More recently, lncRNAs have been associated with biology of GC. However, the prognostic values of lncRNAs in GC have not been clarified clearly.

To identify the prognostic lncRNA genes, we profiled lncRNA by mining the existing microarray gene expression data on a variety of commonly used commercial arrays. Of those, the Affymetrix Human Genome U133 array series is one of the most commonly used commercial microarrays in human cancer profiling [56]. As a public gene expression data repository, GEO has contained lots of gene expression data that could be used for further analysis. Based on this mining method, we additionally applied another method to select prognostic lncRNA genes. Predictors (genes) were selected by applying the random survival forest-variable hunting (RSF-VH) algorithm [36]. The random forests method is classified into a tree-based method which has an advantage in detecting interactions. This algorithm exploits maximal subtrees for effective variable selection, and the trees in a survival forest are grown randomly using a two-step randomization process [36]. Moreover, it has been developed for processing data with several variables larger than the number of samples. There is no denying that many published studies applied univariable and multivariable analyses on microarray data for screening where potential genes interacting with other genes may be dropped from the analyses. Actually, in this regard, the RSF-VH algorithm would be more powerful.

### Functional characteristics of the 24 prognostic lncRNAs

We finally identified a set of 24 lncRNAs that showed differential expressions among the GC patients included in the data sets. Such differentiations signified their potential roles in GC. Although some of these deregulated lncRNAs have been reported to express in cancer or other disorders, they have not been investigated in GC. For example, the expression of AK026189 (CASC 15) was found to be associated with neurobalstoma and was increased during melanoma progression [57–59]. And it was regarded as an independent predictor of disease recurrence in a cohort of 141 patients with AJCC stage III lymph node metastasis [58]. In our study, AK026189 was highly expressed in GC and was found to be correlated with shortened survival. Another candidate, H04858 (MIR99AHG, MONC) was also abundantly expressed in GC samples. A study has revealed that H04858 was highly expressed in acute megakaryoblastic leukemia cell lines serving as a regulator of hematopoiesis and oncogene in the development of myeloid leukemia [60]. Thus, we infer that H04858 may act as an oncogene in GC tumorigenesis and further investigations are great needed as well.

Moreover, lncRNA AI916498 (TRAF3IP2-AS1) was found differentially expressed in midbrain dopamine cells of human cocaine abusers and its transcript showed a surprisingly strong nuclear localization in dopamine cells [61]. Bannon et al [61] suggested that AI916498 might act as a mediator of a disruption of NF-KB signaling seen in cocaine abuse. More interestingly, AI916498 was down-regulated in the human gastric cell lines after received iodine-125 particle irradiation [62]. This indicated that AI916498 may play a critical role in the iodine-125 seed treatment of GC and be a potential target for developing anti-gastric cancer drugs in the future. Also, AA041523 (LINC00473, C6orf176) was first discovered as a regulator of cAMP-mediated gene expression and may serve as a biomarker or a drug target in context of diseases with deregulated cAMP signaling [63]. And further investigation demonstrated that AA041523 mediated decidualization of human endometrial stromal cells and the expression of AA041523 was regulated by cAMP-PKA pathway through IL-11-mediated STAT3 phosphorylation [64]. Recently, the elevated expression of AA041523 was highly associated with loss function of the tumor suppressor LKB1 gene, one of the most common mutational events in lung cancer [65]. Further analysis suggested that AA041523 could act as a biomarker or a therapeutic target for lung cancer with impaired LKB1 signaling [65]. Additionally, AA041523 was found down-regulated in Helicobacter pylori-infected cells which might contribute to the pathological responses and development of Helicobacter pylori related disease [66]. In our study, AA041523 was also highly expressed in GC and involved with shorten survival. Thus, we suggest that AA041523 may play a critical role in GC tumorigenesis. More importantly, BC021187 (DKFZP434K028) got a lower expression level in GC tissues and the low expression was correlated with larger tumor size [67]. In the present study, the higher level of BC021187was associated with longer survival, suggesting a protective role in GC biogenesis. And further investigations are needed to confirm that.

Among the 24 lncRNAs, except for those mentioned above, some lncRNAs were either poorly investigated or have not been reported. For instance, BE621082 (LINC0142) was identified as a novel susceptibility locus on Xp11.21 associated with systemic lupus erythematosus (SLE) [68]. Moreover, as a super enhancer, AL703532 (CARMN) was regarded as a regulator of cardiac cell differentiation and hemeostasis [69]. Additionally, as a gene on the opposite strand of the ghrelin gene, AI056187 (GHRLOS) spanned the promoter and untranslated regions of the ghrelin gene [70] and lowly expressed in the normal gastric tissues [70]. In our study, AI056187 was highly expressed in GC and significantly correlated with longer survival which indicated a protective role in GC biogenesis. As for the rest of the lncRNAs, such as AF035291, AI028608, BC037827, BC038210, AA463827, AK056852, AW206234 and AI095542 were associated with shorten survival in our study, whereas AI080288, BF238392, BC005107, BC039674, T79746, H11436, BF511694, BC035722 were associated with prolonged survival. Although the roles of these genes in GC or other diseases biogenesis are presently unclear, our findings suggest that they deserve further investigations.

### The 24-lncRNA signature is a significant determinant of survival in GC

By applying the 24-lncRNA signature to the GSE62254 test series patients, a clear separation was observed in survival curves between patients with high- and low-risk signatures. Patients with a high-risk 24-lncRNA signature in their tumor specimens tended to have shortened survival, whereas patients with a low-risk 24-lncRNA signature tended to have prolonged survival. The association between the lncRNA signature and survival was significant no matter whether the former was evaluated as a continuous variable or category variable (divided by the median cutoff). The usefulness of this lncRNA signature could be internally validated in the non-overlapping GSE62254 patients (the validation series) and another independent cohort of GSE15459 that profiled through the same platform of GSE62254, indicating good reproducibility of this 24-lncRNA signature in GC. Taken together, our results suggest that the 24-lncRNA signature may be a significant determinant of survival in GC, rather than an accidental feature of the transcription noise.

### The 24-lncRNA signature is an independent prognostic factors in GC

Further analysis uncovered that the prognostic value of the 24-lncRNA signature was independent of one of the main prognostic factors in GC, LNR. LNR was defined as the ratio of the number of metastatic lymph nodes to the number of removed lymph nodes [47]. Recently, LNR has gained increasing attention in researches because of its lymph node status in AJCC TNM stage system [71, 72]. In Japan, LNR has been repeatedly reported to be of prognostic relevance in advanced GC in the multivariate analysis [73]. Two studies have indicated that LNR = 16.7 % was optimal cutoff level as an effective prognostic indictor in advanced GC [46, 47]. Patients with LNR ≥ 16.7 %got a shortened survival than those of LNR <16.7 % [46, 47]. Therefore, it is important to evaluate whether the prognostic value observed on our 24-lncRNA signature is independent of this known strong prognostic factor or not. By performing multivariable Cox regression analysis and LNR stratification analysis, we identified LNR-independent prognostic values of the 24-lncRNA signature in GC patients of the entire GSE62254 data set.

Moreover, the prognostic value of the 24-lncRNA signature was also independent of the postoperative chemotherapy. Currently, surgery followed by chemoradiotherapy is the standard protocol in the United States, whereas perioperative or postoperative chemotherapy is recommended in the Europe and Asia. Increasing meta-analyses published have demonstrated that postoperative chemotherapy could prolong the survival [74, 75]. In the present study, our results indicated that patients with different prognoses could be divided into high- or low- risk group by the 24-lncRNA signature despite of the same postoperative chemotherapy stratum. And this further strongly demonstrated that the 24-lncRNA signature could act as an independent prognostic factor for GC. Finally, it was fascinating to find that the 24-lncRNA signature was almost independent of each TNM stage of AJCC and had a similar survival predictive ability as AJCC stage. Moreover, 24-lncRNA signature combined with AJCC stage had a stronger power for DFS prediction in the ROC analysis. At last, the 24-lncRNA signature may have a better predictive ability than both LNR and postoperative chemotherapy alone. Thus, the ability of our 24-lncRNA signature in identifying subgroups of GC patients with identical AJCC stage implies that the lncRNA signature may be used to refining the current prognostic model and facilitating further stratification of patients in the future clinical trials.

### The implication of the study

The function of lncRNAs were more likely to correlate with their transcript abundance as they do not encode proteins [76]. Actually, lncRNAs have been demonstrated to have higher specificity than protein-coding mRNAs [77, 78]. Our findings may have clinical implications in the development of a novel, independent prognostic factor of GC. Additionally, given the expression of lncRNAs could be handled with transgene approaches such as the lncRNA interference (RNAi)-mediated gene silencing technology, for instance, knock-down of the classical lncRNA HOTAIR using specific siRNAs was indicated to be associated with the metastatic potential of breast cancer cells [54]. Although some of the 24 lncRNAs have not investigated or reported in GC or other diseases, we also have reason to believe that these lncRNAs may contribute to GC biogenesis. Of course, amount of analyses are greatly needed in the future investigations.

### The limitations of the study

The limitations should be acknowledged for our study. First, since the two GEO data sets involved in this study were profiled through Affymetrix Human Genome U133 Plus 2.0 chips which represents part but not all of the possible lncRNA presents, the lncRNAs candidates indentified here may not represent the complete lncRNA populations underlying GC biological behavior. Second, the DFS was regarded as the primary endpoint in the test and internal validation data set (GSE62254). Unfortunately, we could only use OS as the endpoint in external validation data set (GSE15459) because this data set did not contain the information about DFS. Despite this drawback, however, the significant and consistent correlation of the 24-lncRNA signature with OS in external validation data set indicates that it is a potential useful prognostic marker for GC. Finally, we have no experimental data and lack information on the mechanism behind the signature lncRNAs, and experimental studies on these lncRNAs are greatly needed to provide important information to further our understanding of their functional rolesin GC.

## Conclusions

This study presents a powerful lncRNA signature by probing and integrating currently available microarray data. This innovative lncRNA signature showed independence of two main prognostic factors, LNR and postoperative chemotherapy. Also this lncRNA signature may contribute to personalize prediction of GC prognosis and acted as potential biomarkers for GC prognostication. The GSEA analysis suggested that this signature might involve with several cancer recurrence and metastasis associated pathways which supported the DFS predictive ability of the signature. The lncRNA profiling approach described here can also be applied in other cancers and will serve as a useful method for the systematic identification of lncRNA biomarkers in clinical practice. Future investigations will concentrate on the validation of our findings in planned clinical trials and the functional explanation of these lncRNAs.

## Additional files

Additional file 1: Table S1. Clinical characteristics of 492 gastric cancer patients involved in the study. (DOCX 15 kb) Additional file 2: Figure S1. Kaplan-Meier estimates of the disease free survival (DFS) of GEO patients using the 24-lncRNA signature, stratified by TNM stage. Entire GSE62254 set (*N =* 300) were first stratified by low TNM stage (I & II) and high TNM stage (III & IV). Kaplan-Meier plots were then used to visualize the survival probabilities for the high-risk versus low-risk group of patients determined on the basis of the median risk score from the GSE62254 set patients. (A) Kaplan-Meier curves for the entire GSE62254 set patients (*N =* 300); (B) Kaplan-Meier curves for patients with low TNM stage (*N =* 127); (C) Kaplan-Meier curves for patients with high TNM stage (*N =* 173). The tick marks on the Kaplan-Meier curves represent the censored subjects. The differences between the two curves were determined by the two-sided log-rank test. (EPS 1031 kb) Additional file 3: Table S2. 24-lncRNA signature related signaling pathways with positive and negative enrichment score ranked by enrichment score. (XLS 267 kb)

## Abbreviations

AJCC: American joint committee on cancer
AUROC: Area under receiver operating characteristic
CI: Confidence interval
DFS: Disease free survival
GC: Gastric cancer
GEO: Gene expression Ominus
GSEA: Gene set enrichment analysis
HR: Hazard ratio
lncRNAs: Long non-coding RNAs
LNR: Lymph node ratio
OS: Overall survival
ROC: Receiver operating characteristic
UICC: Union international committee on cancer
