# Supplementary material for: A long non-coding RNA signature to improve prognosis prediction of gastric cancer
Source: Mol Cancer. 2016 Sep 20;15:60. doi: 10.1186/s12943-016-0544-0 (PMC5029104; doi:10.1186/s12943-016-0544-0)
Supplement: Additional file 1: Table S1. — Clinical characteristics of 492 gastric cancer patients involved in the study. (DOCX 15 kb) [file 12943_2016_544_MOESM1_ESM.docx]

**Table S1. Clinical characteristics of 492 gastric cancer patients involved in the study.**

| **Features** | **Low-risk Group** | **High-risk group** | ***P* value** |
| --- | --- | --- | --- |
| **GSE62254 test series(N=180)** | **N=90** | **N=90** |  |
| Age(mean+SD) | 63.8+10.7 | 60.4+12.4 | 0.049^a^ |
| Gender |  |  |  |
| Female | 27(30.00%) | 37(41.11%) | 0.119^b^ |
| Male | 63(70.00%) | 53(58.89%) |  |
| TNM stage |  |  |  |
| I | 11(12.22%) | 4(4.44%) | 0.009^b^ |
| II | 37(41.11%) | 23(25.56%) |  |
| III | 20(22.22%) | 36(40.00%) |  |
| IV | 22(24.44%) | 27(30.00%) |  |
| **GSE62254 validation series(N=120)** | **N=60** | **N=60** |  |
| Age(mean+SD) | 60.2+10.3 | 63.2+11.5 | 0.141^a^ |
| Gender |  |  |  |
| Female | 19(31.67%) | 18(30.00%) | 0.843^b^ |
| Male | 41(68.33%) | 42(70.00%) |  |
| TNM stage |  |  |  |
| I | 11(18.33%) | 4(6.67%) | 0.054^b^ |
| II | 22(36.67%) | 15(25.00%) |  |
| III | 15(25.00%) | 25(41.67%) |  |
| IV | 12(20.00%) | 16(26.67%) |  |
| **GSE15459 series(N=192)** | **N=96** | **N=96** |  |
| Age(mean+SD) | 66.0+12.5 | 62.8+13.8 | 0.096^a^ |
| Gender |  |  |  |
| Female | 28(29.17%) | 39(40.63%) | 0.096^b^ |
| Male | 68(70.83%) | 57(59.38%) |  |
| TNM stage |  |  |  |
| I | 21(21.88%) | 10(10.42%) | 0.064^b^ |
| II | 15(15.63%) | 14(14.58%) |  |
| III | 37(38.54%) | 35(36.46%) |  |
| IV | 23(23.96%) | 37(38.54%) |  |

^a^Student’s test; ^b^Chi-square test.
